# Supplementary material for: Development and Integration of Patient-Reported Measures into E-Health System: Pilot Feasibility Study
Source: Healthcare (Basel). 2023 Aug 14;11(16):2290. doi: 10.3390/healthcare11162290 (PMC10454584; doi:10.3390/healthcare11162290)
Supplement: Supplementary file 1 [file healthcare-11-02290-s001.zip › Supplement 1_PREM Questionnaire.pdf]

Supplement 1. Original patient-reported experience measure (PREM) instrument

1. My family doctor is involved in the treatment of my illness. Family doctor(s) and nurse(s) are familiar with my illness, can provide requested information and help.
2. The process from onset of my symptoms to diagnosis was smooth. When I felt the first symptoms of the illness, I had no problem getting to my family doctor or to the speciality doctor and perform diagnostic tests.
3. The environment of the primary care doctor facilities is patient-friendly. The staff at the primary care is friendly, understanding of my needs, and is always ready to provide help. I did not experience negative attitude, bullying, the environment of the primary care is adapted to make the patient feel pleasurable.
4. The environment of the outpatient facilities at Vilnius University Hospital Santaros Clinics is patient-friendly. The staff of the outpatient facilities is friendly, understands patient's needs and is always ready to provide assistance. I did not experience negative attitude, bullying, the environment of the polyclinic is adapted to make the patient feel pleasurable.
5. The environment of the Vilnius University Hospital Santaros Clinics, day-care facilities is patient-friendly. The staff of the day care is friendly, trying to understand patient's needs and is always ready to provide assistance. I did not experience negative attitude, bullying, the environment of the inpatient facility is adapted to make the patient feel pleasurable.
6. I have not encountered any technical problems in the provision of healthcare services. I have had no problems with referrals, prescribing and obtaining reimbursed medications.
7. I received enough information about my illness. I was provided enough information about the disease and its treatment.
8. The environment of the hospital in-patient facilities is patient-friendly. The hospital staff is friendly, understanding of my needs and are always ready to provide help. I did not experience negative attitude, bullying, and the hospital environment is designed to make the patient feel pleasurable.
9. I was offered professional psychological help. During treatment at the hospital, I received an offer of referral for professional help from a psychologist.
10. I received sufficient information about existing social services. After diagnosis and during treatment, I received sufficient information about all additional social services, incapacity for work, the procedure for granting disability and other support options.
11. I received enough information on continuing treatment at home. I was provided with clear information about further treatment or monitoring strategy, recommendations for staying at home, acquiring prescribed medications, contact information.
12. Rehabilitation treatment. I was treated as good as possible in a sanatorium or rehabilitation facility to recover from medical treatment and recuperate.

Five-level responses were provided: 1 – fully agree; 2 –agree; 3 – neither agree nor disagree; 4 – disagree; 5 – fully disagree.
