# Supplementary material for: Development and Integration of Patient-Reported Measures into E-Health System: Pilot Feasibility Study
Source: Healthcare (Basel). 2023 Aug 14;11(16):2290. doi: 10.3390/healthcare11162290 (PMC10454584; doi:10.3390/healthcare11162290)
Supplement: Supplementary file 1 [file healthcare-11-02290-s001.zip › Supplement 2_Item-item and item-total correlations of PROM.pdf]

Supplement 2. Item-item and item-total correlations of patient-reported outcomes measure questionnaire responses.

		Spearman's Correlation Coefficient (ϱ)	*p*
**Dimensions**			
Mobility	Self-care	0.748	<0.001
Mobility	Usual activities	0.702	<0.001
Mobility	Pain/discomfort	0.682	<0.001
Mobility	Anxiety/depression	0.484	<0.001
Mobility	VAS	−0.620	<0.001
Self-care	Usual activities	0.683	<0.001
Self-care	Pain/discomfort	0.534	<0.001
Self-care	Anxiety/depression	0.403	<0.001
Self-care	VAS	−0.522	<0.001
Usual activities	Pain/discomfort	0.630	<0.001
Usual activities	Anxiety/depression	0.371	<0.001
Usual activities	VAS	−0.616	<0.001
Pain/discomfort	Anxiety/depression	0.481	<0.001
Pain/discomfort	VAS	−0.585	<0.001
Anxiety/ depression	VAS	−0.482	<0.001
**Dimension with a total score**			
Mobility	EQ-5D total score	−0.870	<0.001
Self-care	EQ-5D total score	−0.743	<0.001
Usual activities	EQ-5D total score	−0.836	<0.001
Pain/discomfort	EQ-5D total score	−0.863	<0.001
Anxiety/ depression	EQ-5D total score	−0.592	<0.001
VAS	EQ-5D total score	0.675	<0.001

VAS—visual analog scale
