# Supplementary material for: Development and Integration of Patient-Reported Measures into E-Health System: Pilot Feasibility Study
Source: Healthcare (Basel). 2023 Aug 14;11(16):2290. doi: 10.3390/healthcare11162290 (PMC10454584; doi:10.3390/healthcare11162290)
Supplement: Supplementary file 1 [file healthcare-11-02290-s001.zip › Supplement 3_Item-item and item-total correlations of PREM.pdf]

Supplement 3. Item-item and item-total correlations of patient-reported experience measure questionnaire responses.

		**Spearman's Correlation Coefficient (ϱ)**	***p***
	**Items**		
Q1	Q2	0.495	<0.001
Q1	Q3	0.368	<0.001
Q1	Q4	0.220	0.003
Q1	Q5	0.120	0.103
Q1	Q6	0.226	<0.001
Q1	Q7	0.319	<0.001
Q1	Q8	0.224	<0.001
Q1	Q9	0.100	0.112
Q1	Q10	0.317	<0.001
Q1	Q11	0.241	<0.001
Q1	Q12	0.083	0.463
Q2	Q3	0.318	<0.001
Q2	Q4	0.436	<0.001
Q2	Q5	0.381	<0.001
Q2	Q6	0.282	<0.001
Q2	Q7	0.379	<0.001
Q2	Q8	0.318	<0.001
Q2	Q9	0.120	0.056
Q2	Q10	0.437	<0.001
Q2	Q11	0.370	<0.001
Q2	Q12	0.208	0.062
Q3	Q4	0.299	<0.001
Q3	Q5	0.332	<0.001
Q3	Q6	0.399	<0.001
Q3	Q7	0.382	<0.001
Q3	Q8	0.325	<0.001
Q3	Q9	0.220	0.001
Q3	Q10	0.390	<0.001
Q3	Q11	0.367	<0.001
Q3	Q12	0.355	0.002
Q4	Q5	0.688	<0.001
Q4	Q6	0.257	<0.001
Q4	Q7	0.453	<0.001
Q4	Q8	0.572	<0.001
Q4	Q9	0.290	<0.001
Q4	Q10	0.427	<0.001
Q4	Q11	0.469	<0.001
Q4	Q12	0.558	<0.001
Q5	Q6	0.314	<0.001
Q5	Q7	0.492	<0.001
Q5	Q8	0.496	<0.001
Q5	Q9	0.259	<0.001
Q5	Q10	0.311	<0.001

Q5	Q11	0.414	<0.001
Q5	Q12	0.291	0.024
Q6	Q7	0.457	<0.001
Q6	Q8	0.426	<0.001
Q6	Q9	0.190	0.002
Q6	Q10	0.161	0.010
Q6	Q11	0.360	<0.001
Q6	Q12	0.197	0.079
Q7	Q8	0.550	<0.001
Q7	Q9	0.255	<0.001
Q7	Q10	0.394	<0.001
Q7	Q11	0.603	<0.001
Q7	Q12	0.375	<0.001
Q8	Q9	0.315	<0.001
Q8	Q10	0.374	<0.001
Q8	Q11	0.522	<0.001
Q8	Q12	0.444	<0.001
Q9	Q10	0.435	<0.001
Q9	Q11	0.378	<0.001
Q9	Q12	0.468	<0.001
Q10	Q11	0.493	<0.001
Q10	Q12	0.345	0.002
Q11	Q12	0.761	<0.001
**Items with a total score (PREM)**			
Q1	PREM total score	0.515	<0.001
Q2	PREM total score	0.620	<0.001
Q3	PREM total score	0.590	<0.001
Q4	PREM total score	0.639	<0.001
Q5	PREM total score	0.562	<0.001
Q6	PREM total score	0.533	<0.001
Q7	PREM total score	0.665	<0.001
Q8	PREM total score	0.664	<0.001
Q9	PREM total score	0.582	<0.001
Q10	PREM total score	0.729	<0.001
Q11	PREM total score	0.735	<0.001
Q12	PREM total score	0.749	<0.001

Q1 to Q12—Question 1 to Question 12 of the original Patient-Reported Experience Measure (PREM). Full questionnaire consisting of 12 questions is provided as Supplement 1.
